# The Frank–Starling Law: a jigsaw of titin proportions

**DOI:** 10.1007/s12551-017-0272-8

**Published:** 2017-06-21

**Authors:** Vasco Sequeira, Jolanda van der Velden

**Affiliations:** 1 Department of Physiology, Institute for Cardiovascular Research, VU University Medical Center, O|2 building, 11W-53, De Boelelaan 1118, 1081 HV Amsterdam, the Netherlands; 2 ICIN- Netherlands Heart Institute, Utrecht, The Netherlands

**Keywords:** Frank-Starling, Titin, Cross-bridges, Ca^2+^-cooperativity

## Abstract

The Frank–Starling Law dictates that the heart is able to match ejection to the dynamic changes occurring during cardiac filling, hence efficiently regulating isovolumetric contraction and shortening. In the last four decades, efforts have been made to identify a common fundamental basis for the Frank–Starling heart that can explain the direct relationship between muscle lengthening and its increased sensitization to Ca^2+^. The term ‘myofilament length-dependent activation’ describes the length-dependent properties of the myofilaments, but what is(are) the underlying molecular mechanism(s) is a matter of ongoing debate. Length-dependent activation increases formation of thick-filament strongly-bound cross-bridges on actin and imposes structural–mechanical alterations on the thin-filament with greater than normal bound Ca^2+^. Stretch-induced effects, rather than changes in filament spacing, appear to be primarily involved in the regulation of length-dependent activation. Here, evidence is provided to support the notion that stretch-mediated effects induced by titin govern alterations of thick-filament force-producing cross-bridges and thin-filament Ca^2+^-cooperative responses.

## The Frank–Starling Law

Building on the work of their German and British predecessors, Otto Frank and Ernest Starling at the turn of the twentieth century described the relationship between the length of the heart fibers and its power of contraction, the so-called Frank–Starling Law of the Heart (Sequeira and van der Velden 2015). This relationship enables beat-to-beat adjustment of cardiac output in response to changes in venous pressure. At the single cardiac cell level, the Law mandates that there is a direct relationship between myofilament length and their sensitivity to Ca^2+^ ions, such that more force is generated at a given concentration of Ca^2+^ as fibers are lengthened. The term ‘myofilament length-dependent activation’ describes the length-dependent properties of the myofilaments. It has been attributed to structural–mechanical alterations of the thin-filament with greater than normal bound Ca^2+^ and to the increased formation of strongly-bound cross-bridges on actin, following muscle stretching (Hibberd and Jewell 1982; Allen and Kentish 1985; Sequeira and van der Velden 2015). Moreover, myofilament length-dependent activation has been shown to be additionally, and importantly, regulated by the degree of contractile protein phosphorylation (Konhilas et al. 2003; Hanft et al. 2013; Sequeira et al. 2013; Wijnker et al. 2014).

## The role of interfilament spacing

Research on interfilament lattice spacing suggested it played a primary role in myofilament length-dependent activation. This concept dominated the literature for two decades until the late 1990s. Evidence emerged that supported the notion that myocyte lengthening was accompanied by an increased sensitization of the thin-filaments to Ca^2+^, and that a reduction in the distance between the myosin cross-bridges and actin filaments facilitated actomyosin interaction. In 1977, Godt and Maughan (1977) used the high-molecular-weight polymer (dextran) to reduce the interfilament distance, thereby varying maximum tension levels of Ca^2+^-activated skeletal muscle fibers. Because dextran was unable to diffuse between the myofilaments, it osmotically compressed them, thereby decreasing the distance of their lattice.

These results were confirmed in consecutive studies, and by other groups, using either skeletal (Godt and Maughan 1981; Moss et al. 1983; Martyn and Gordon 1988; Wang and Fuchs 1995) or cardiac (McDonald and Moss 1995; Wang and Fuchs 1995) muscle preparations, adding support to the idea that lattice spacing, rather than changes in length, was responsible for the changes in myofilament Ca^2+^-sensitivity. These findings were in good agreement with the proposition of Hofmann and Fuchs (1987a, b) and Fuchs and Wang (1996) who showed that both Ca^2+^-sensitivity and Ca^2+^-affinity to cardiac troponin C (cTnC) were directly correlated with changes of lattice spacing but not with sarcomere length in cardiac muscle.

## Is Ca^2+^-sensitivity directly related to changes in myofilament lattice?

Despite the large amount of evidence supporting the lattice spacing mediated-effects as the primary mechanism involved in length-dependent activation, filament lattice alterations were never directly measured when dextran was added to the muscle preparations. Instead, indirect assessment was based on the assumption that lattice alterations nearly correlated with changes of muscle width. Direct visualization of changes to interfilament lattice spacing as a function of dextran application, was published in 2000–2003 by the Pieter de Tombe laboratory (Irving et al. 2000; Konhilas et al. 2002b, 2003), who used synchrotron X-ray diffraction in membrane-permeabilized and intact cardiac tissue. They showed that myofilament Ca^2+^-sensitivity was not linearly related to changes in interfilament spacing as a result of osmotic compression by dextran. Instead, compression of the lattice, observed when the sarcomere length was increased to optimal length (2.2 μm), did not affect myofilament Ca^2+^-sensitivity (Konhilas et al. 2002b, 2003). Konhilas et al. (2002b, 2003) suggested that lattice changes are at least secondary in length-dependent activation.

## How does stretch activate contraction?

If not by lattice spacing alterations, how does stretching or increasing fiber length govern length-dependent activation and the Frank–Starling effect? What is at the heart of the Frank–Starling “jigsaw”? Overall, not one, but several, synergistically mechanisms, mediated by the giant protein titin following fiber stretch, appear to drive enhanced Ca^2+^ bound to cardiac troponin C (cTnC). The formation of increased numbers and/or more strongly-bound cross-bridges on actin increase the cooperation of force-producing thin-filament units. Stretch-induced effects, rather than changes in filament spacing, mediated by titin, appear to be primarily involved in the regulation of length-dependent activation. In this review, evidence is presented that supports the idea that upon stretch—because it is anchored to the Z-disk–titin can not only act on thick-filament structures (and therefore is in a good position to orchestrate direct changes on cross-bridges) but also on thin-filament functional units (actin-tropomyosin-troponin).

## Titin-mediated recruitment of thick-filament cross-bridges

Evidence from the last decade revealed that the giant elastic protein titin may serve as a length-dependent mechanosensor. Titin is a huge (3.8 MDa) protein that extends from the Z-disc to the M-band (Maruyama et al. 1985; Fürst et al. 1988). Its N-terminal region interacts with actin in the Z-disc (Trombitás et al. 1997). The I-band region of titin is extensible and consists of three elastic components that act as a spring (Labeit and Kolmerer 1995). In the A-band, titin is relatively inextensible because it interacts with the thick-filament proteins, such as the light meromyosin portion of myosin and cardiac myosin-binding protein C (cMyBP-C). Titin then links up with titin in the opposite half-sarcomere at the M-line (Maruyama et al. 1985; Freiburg and Gautel 1996; Zoghbi et al. 2008). Titin may exert its effects in two ways. Its primarily effect seems to involve thick-filament cross-bridge recruitment, and its secondary effect is to decrease the filament lattice to approximate myosin and actin filaments.

**Fig. 1:**
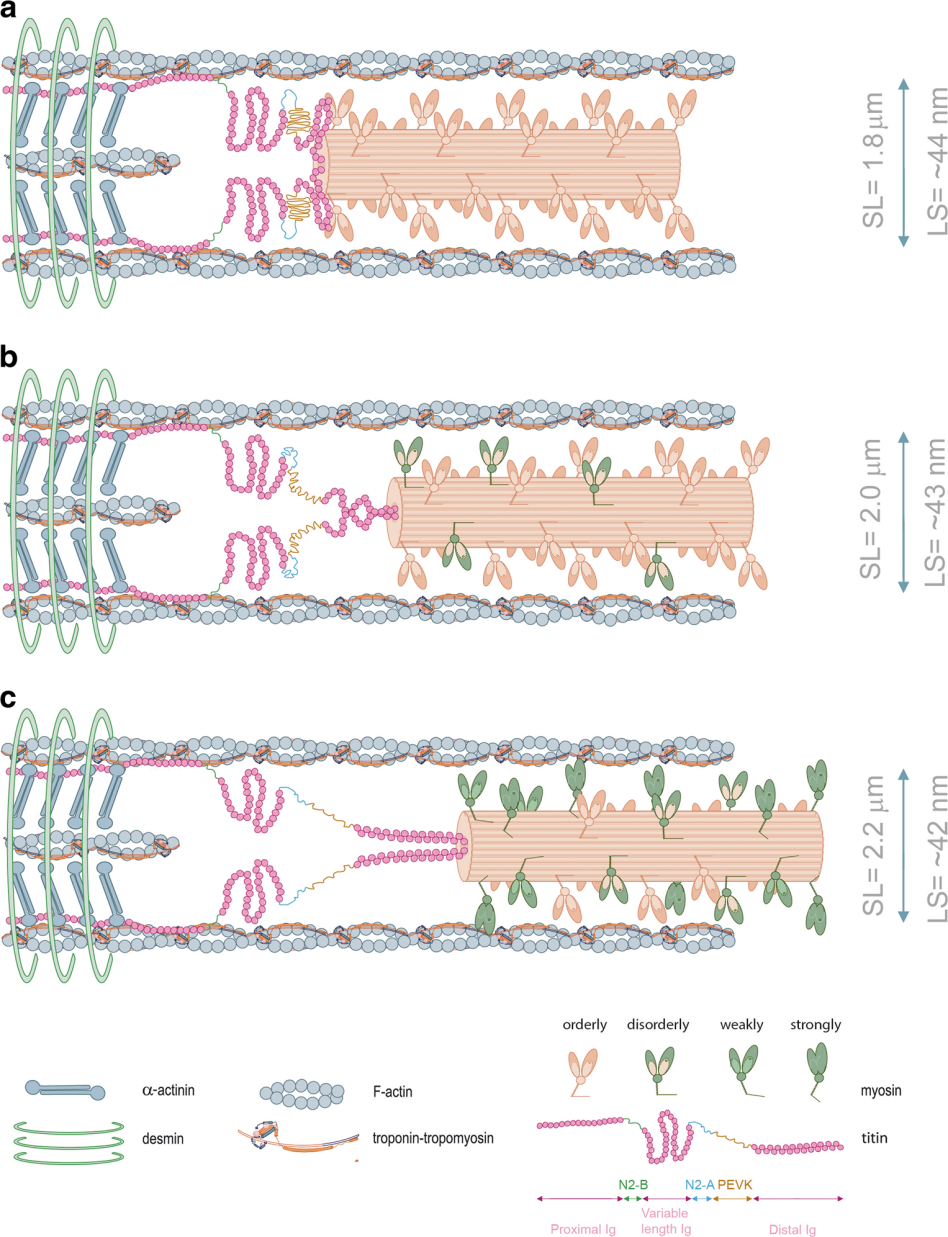
A schematic model of half-sarcomere at varying sarcomere lengths at low Ca^2+^ conditions. Lattice spacing dimensions at each varying length were taken from Konhilas et al. (2002a). As the muscle is stretched from a relatively short sarcomere length (**a**) to higher sarcomere lengths (**b**, **c**), lattice spacing becomes smaller, myosin approximates to actin and cross-bridges transit from orderly into disorderly states. The I-band region of titin is the extensible region and consists of three elastic components that act as a spring element: (1) tandem immunoglobulin (Ig)-like domain regions, with proximal (near Z-disc) and distal (near I-A regions) segments; (2) the PEVK sequence-region rich in proline (P), glutamic acid (E), valine (V) and lysine (K); and (3) the N2B and N2BA elements (both isoforms contain N2B segments, but only the N2BA isoform contains an additional N2A element) (Labeit and Kolmerer 1995). Titin-induced stretch imposes a passive strain on the thick-filament proteins, which reduces lattice spacing and changes the arrangement of cross-bridges. Upon fiber lengthening, titin anchorage both on actin and tropomyosin at the I-band region, imposes a passive strain on thin-filament proteins and increases the cooperative unit size. Please note that more myosin motors are turned “On” during systolic activation (Reconditi et al. 2017). Distinct myosin colors are depicted to better illustrate the transition of orderly to disorderly projections. α-actinin and desmin illustrate the Z-disc border. Note: cardiac myosin-binding protein C (cMyBP-C) was omitted to simplify the drawing and the width and sarcomere length dimensions are not to scale. (image adapted from Sequeira and van der Velden 2015)

Stretch appears to exert its titin-dependent length activation by potentiating the recruitment of rested-to-ready strongly-bound cross-bridges. X-ray structural studies has provided evidence that diastolic lengthening rearranges thick-filament structures, such that more cross-bridges are recruited for activation (Ait-Mou et al. 2016). Increased cross-bridge recruitment is directly associated with myofilament length-dependent activation (Ait-Mou et al. 2016). Because titin is obliquely oriented to the sarcomere axis and attaches both myosin and cMyBP-C (Maruyama et al. 1985; Freiburg and Gautel 1996; Zoghbi et al. 2008), it will impose a passive strain on thick-filament proteins, thereby changing the geometry of cross-bridges (Fukuda et al. 2000, 2001; Ait-Mou et al. 2016). In frog skeletal muscle, sarcomere lengthening increases myosin periodicity (Wakabayashi et al. 1994), such that the transition of the population of myosin cross-bridges from rested (“Off”) to weakly-bound increases (i.e., from an orderly to a disorderly state with respect to the axis of the thick-filament) (Fig. 1) (Malinchik et al. 1997; Xu et al. 1997). A similar finding is observed in cardiac muscle where the orientation of myosin heads becomes more perpendicular to the thick-filament axis when sarcomere length is increased (i.e., the proportion of cross-bridge motors switched “On” increases upon stretch) (Farman et al. 2011). These studies suggest that stretch-induced activation by titin imposes a radial strain over the thick-filament, which recruits rested-to-ready force-generating cross-bridges under low Ca^2+^-activation conditions (Fig. 1). The magnitude of this effect is dependent on the rise of systolic stress, such that, at high filament loads during systolic activation, this mechanosensing ability recruits more force-producing cross-bridges (Linari et al. 2015; Reconditi et al. 2017). In other words, the fraction of “super relaxed” cross-bridge units transiting from the Off to the On state increases with thick-filament stress (Kampourakis et al. 2016; Reconditi et al. 2017).

Although a reduced filament lattice spacing may not be primarily involved in length-dependent activation, one cannot entirely exclude the prospect that lattice spacing reductions may secondarily exacerbate the effects of fiber lengthening (Fig. 1). An increase in titin-based passive tension reduces filament lattice upon stretch and approximates myosin and actin. A correlation between enhanced length-dependent activation and higher levels of passive tension has been reported (Cazorla et al. 1999, 2001; Fukuda et al. 2003). The concept is also supported by a recent report using a rat model expressing a giant titin isoform, which clearly associates reduced myofilament force development and impaired length-dependent activation (Mateja et al. 2012). Hence, reductions in the titin-induced changes in lattice bring the cross-bridges closer to the thin-filaments, thus favoring force development of Ca^2+^-activated fibers.

## Titin-induced strain on the thick-filament increases Off-On transition of thin-filament units

Length-dependent activation has been associated with titin-induced strain on the thick-filament and the subsequent potentiation of Off–On transition of thin-filament units (the steric blocking–unblocking model) (Terui et al. 2008; Farman et al. 2010; Mateja et al. 2012). There is evidence to suggest that length-dependent activation is regulated via an Off–On switch of the thin-filament, potentiated by the greater movement of tropomyosin on actin that uncovers more myosin-binding sites (Smith and Fuchs 1999). In the absence of Ca^2+^ (B-state), tropomyosin sterically blocks the myosin-binding sites on actin. In cardiomyocytes, a rise in Ca^2+^ induces a conformational change in the troponin–tropomyosin complex, resulting in the movement of tropomyosin on actin which exposes most of its myosin-binding sites (C-state). Weakly-bound cross-bridges populate the C-state (myosin-ADP-Pi). Transition to the myosin-induced state and muscle activation (M-state) involves the release of Pi from cross-bridges and the formation of strong-binding cross-bridges (myosin-ADP). This induces additional movement of tropomyosin, resulting in contraction and myofilament sliding. The relevance of the transition from the B- to the C-state for proper length-dependent activation was shown more than two decades ago by Smith and Fuchs (1999), who were the first to provide evidence for a length-sensitive step in the Off–On transition of thin-filament units. A reduction in ionic strength (<0.05 M) was known to shift the B- to the C-state equilibrium (where the population of recruited cross-bridges increases; Xu et al. 1987; Head et al. 1995) coincided with impaired length-dependent activation (Smith and Fuchs 1999). Terui et al. (2008) subsequently demonstrated that length-dependent activation is associated with titin-induced strain on the thick-filament and the troponin complex. They reconstituted cardiac thin-filaments with fast skeletal troponin and observed reduced length-dependent activation to levels similar to that of skeletal muscle. In turn, reconstitution with cTn restored length-dependent activation, which they associated with increased transition from the B- to the C-state (Terui et al. 2008).

## Similarities to insect flight muscle

Recent findings from stretch-activation of insect flight muscle support the view that Off–On thin-filament transitions are central to the length-dependent response. Activation of these muscles requires a stretch-activation mechanism in addition to Ca^2+^. Because stretching increases the periodicity of myosin (and a re-orientation of myosin heads on the thick-filament; Wakabayashi et al. 1994; Farman et al. 2011), insect flight muscle activation also seems to require the formation of strongly-bound cross-bridges recruited by stretch and Ca^2+^-binding to TnC, in order to potentiate full movement of tropomyosin and accessibility of myosin-binding sites. In support, Perz-Edwards et al. (2011) recently confirmed this proposition by demonstrating that stretch-activation of insect flight muscle requires the steric blocking–unblocking model (Off–On thin-filament transition), such that strong-binding cross-bridges are secondarily recruited to induce the movement of tropomyosin. This study is also interesting because the authors observed X-ray reflections consistent with the existence of “troponin-bridges”, i.e., a combination of cross-bridges bound to the troponin complex. Thus, the similarities between insect flight and vertebrate muscle leave us with the likelihood that passive strain imposed onto the thick-filament of cardiac muscle increases the “activation” of ready-to-weak cross-bridges with direct troponin-bridge formation (and/or even tropomyosin; Behrmann et al. 2012; actomyosin-troponin-tropomyosin bridges). The greater the stretch, the greater the cross-bridge recruitment, and hence a magnified length-dependent response.

## How does increased myosin-binding to actin potentiate greater than normal bound Ca^2+^ on cTnC?

Strong-binding cross-bridges appear to promote global conformational changes in the N-lobe of cTnC (Dong et al. 2007). By reconstitution of myofilaments with labeled cTnC, Dong et al. (2007) measured changes on the cTnC structure when myosin binds. Their results support the concept that strongly-bound myosin is required to open the structure of the N-lobe of cTnC thereby increasing myofilament Ca^2+^-sensitivity. This is consistent with earlier observations that show that Ca^2+^ is released when muscle fibers are shortened and deactivated, implying an active process of cross-bridge detachment and regulation of the Ca^2+^-affinity of cTnC. Allen and Kurihara (1982) microinjected the Ca^2+^-luminescent indicator aequorin in isolated papillary and trabeculae muscles, and observed a rise in intracellular [Ca^2+^] following a quick step-release during contraction, which they attributed to dissociation of Ca^2+^ from the contractile proteins. Housmans et al. (1983) observed similar phenomena. This view was further supported in membrane-permeabilized muscle preparations by Allen and Kentish (1988) who concluded that Ca^2+^ was actively released from the contractile apparatus. Hofmann and Fuchs (1987b, 1988) also showed that length-dependent changes affect the Ca^2+^-affinity of cTnC, by reducing the Ca^2+^-binding affinity of cTnC upon a decrease in sarcomere length. Taken together, these studies indicate that, following fiber lengthening (or shortening), cross-bridge recruitment increases (or decreases) and this potentiates troponin conformational alterations with more (or less) bound Ca^2+^; arguably, these can be attributed to the mechanosensing role of titin in length-dependent increases (or decreases) in force.

More recently, we have provided evidence that human sarcomeric thin-filament mutations disrupt thin-filament transitions with greater accessibility of myosin-bringing sites on actin, and these are associated with reduced length-dependence and myofilament Ca^2+^-sensitization (Sequeira et al. 2013, 2015). It is worth mentioning that a recent study from Dvornikov et al. (2016) puts into question whether thin-filament mutations directly impact myofilament length-dependent activation. Specifically, they found that the cardiac troponin I (cTnI) R145W mutation (known to cause hypertrophic and restrictive cardiomyopathy) does not (directly) disrupt length-dependent activation. Instead, the authors observed that the cTnI R145W mutation directly impairs cTnI-phosphorylation of the two serine sites in humans (serines 23 and 24) responsible for the regulation of length-dependent activation (Dvornikov et al. 2016). The discrepancy in their results is unclear, since, in our studies using human material from a patient with the cTnI R145W mutation (and incubating the cardiomyocytes with Protein Kinase A to exogenously phosphorylate cTnI-S23/S24, validated by specific antibody staining), we did find that the cTnI R145W mutation has the ability *by* *itself* to impair length-dependent activation (Sequeira et al. 2013).

## Titin-induced strain on Tropomyosin’s stiffness increases the Off-On transition of thin-filament units

The thin-filament functional unit comprises seven actin monomers spanned by one tropomyosin dimer and one cTn complex (A_7_TmTn) (Huxley 1973). Ca^2+^-binding to cTnC promotes cTnI detachment from actin and potentiates tropomyosin movement, resulting in the exposure of myosin-binding sites. This movement allows a Ca^2+^-cooperative activation of the thin-filament with additional recruitment of strong-binding cross-bridges. Structural data suggest that each individual strongly-bound cross-bridge binds to a single regulatory unit (A_7_TmTn spanning ∼38.5 nm), and may regulate tropomyosin movement for up to about three units (covering ∼115 nm) along the thin-filament in the presence of Ca^2+^ (Vibert et al. 1997). This has been validated in biochemical studies (Geeves and Lehrer 1994; Maytum et al. 1998).

Tropomyosin increases *communication* between near-neighboring regulatory units, a property governed by the head-to-tail interaction of tropomyosin (i.e., overlap region) (Hill et al. 1980; Nagashima and Asakura 1982; Heeley et al. 1989; Pan et al. 1989; Geeves and Lehrer 1994). Removal of this overlap reduces cooperative-binding of myosin (Johnson and Smillie 1977; Heeley et al. 1989; Pan et al. 1989). Two recent studies support the idea that Ca^2+^-cooperative effects are independent of myosin-binding and are strongly associated with the thin-filaments (Sun et al. 2009; Farman et al. 2010). Sun et al. (2009) reconstituted cardiac thin-filaments with fluorescent-labeled TnC and analyzed changes in the orientation of the structure of troponin. They observed that blebbistatin (which prevents strong-binding formation) had no effect on the Hill coefficient (Sun et al. 2009). Farman et al. (2010) reached similar conclusions, because, in their experiments, blebbistatin decreased Ca^2+^-sensitivity and force, but did not affect the Hill coefficient. Importantly, the authors reconstituted rat cardiac thin-filaments with a cTnC mutant incapable of binding Ca^2+^, and observed that both Ca^2+^-sensitivity and the Hill coefficient were decreased (Farman et al. 2010). They also observed that the effects of the cTnC mutant were greater at shorter (2.0 μm) than at longer (2.2 μm) sarcomere lengths. The later studies are consistent with recent X-ray structural investigations that also support troponin thin-filament rearrangements upon diastolic lengthening, independent of thick-filament alterations (Ait-Mou et al. 2016). Specifically, Ait-Mou et al. (2016) found that, aside from myosin-strain alterations induced by titin, troponin alterations were also shown to be strain-dependent, and their results support both mechanisms (troponin- and myosin-strain dependency) operating simultaneously upon myofilament stretch.

In the study of Farman et al. (2010), the authors attributed thin-filament length-dependent activation due to the ability of tropomyosin to recruit more regulatory units (A_7_TmTn) upon stretch. Because of the tight crosstalk between troponin–tropomyosin interactions, the increased stiffness of tropomyosin at longer sarcomere lengths affects three to four A_7_TmTn units in tandem. Conversely, at shorter muscle lengths, tropomyosin is “weakly” stiff, and hence affects fewer thin-filament units (i.e., less troponin structural rearrangements).

**Fig. 2:**
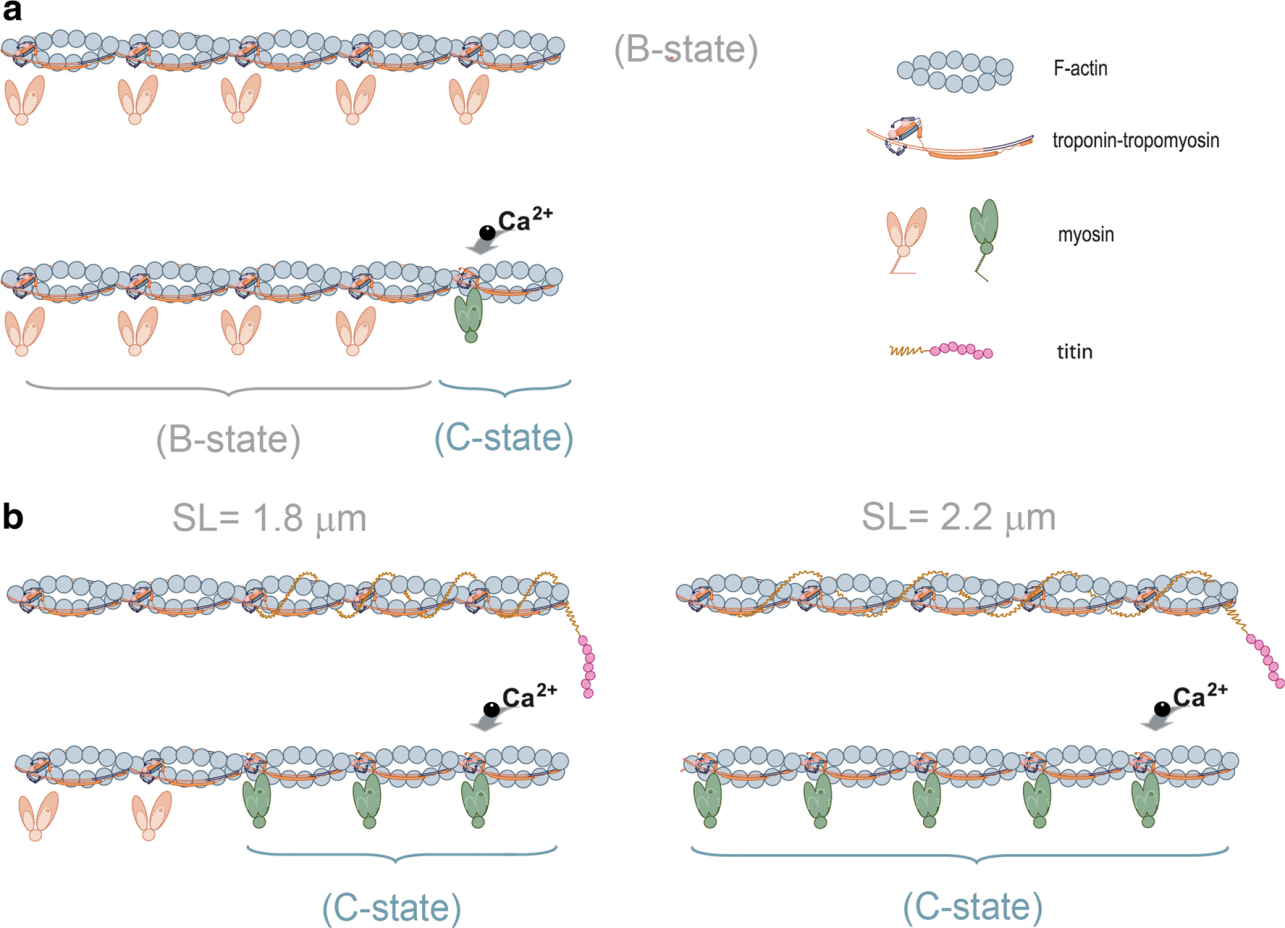
Proposed scheme for tropomyosin-induced Ca^2+^-cooperative activation induced by titin. **a** In the absence of Ca^2+^ (B-state), tropomyosin sterically blocks the myosin-binding sites on actin. Upon Ca^2+^ rise, one functional unit comprising of seven actin monomers spanned by one tropomyosin dimer and one cTn complex (A_7_TmTn) are activated, resulting from Ca^2+^-binding to cTnC which leads to tropomyosin movement and the binding of a single weakly-bound cross-bridge (C-state). **b**, *left* At shorter sarcomere lengths, tropomyosin-induced stretch by titin increases tropomyosin’s stiffness, leading to Ca^2+^-cooperative activation (C-state transition) of up to two additional near-neighboring functional units. **b**, *right* At longer sarcomere lengths, an even stiffer tropomyosin induced via greater titin-passive strain at the thin-filaments, additionally activates up to four functional units. Greater amounts of myosin-binding sites are available at longer muscle lengths. This coincides with both the increased transition of ready to weakly-bound cross-bridges imposed by titin at the thick-filament and the approximation of myosin and actin due to reductions of lattice spacing

When considering how length-dependent alterations influence the cooperative unit size of tropomyosin (presumably via changes in stiffness) by an independent cross-bridge component, we suggest that titin may be responsible (Fig. 2). It not only binds to actin at the Z-disc (Trombitás et al. 1997) but also binds both thin-filaments (actin and tropomyosin) in the I-band region (Raynaud et al. 2004). We *propose* that, upon stretch, titin-binding to tropomyosin at the I-band magnifies the stiffness of tropomyosin’s, thereby increasing the functional unit size, independent on its action of cross-bridges (Fig. 2). We recognize that such a mechanism would allow titin to mediate thin-filament activation by affecting the Off–On transition of tropomyosin, troponin structure alterations, and the Ca^2+^-cooperative size (Fig. 2).

## Tryptic digestion of titin

An interesting result from Fukuda et al. (2001, 2005), which shares similarities with the study of Farman et al. (2010), may provide some support to this idea. The authors observed that trypsin digestion of titin does not affect shorter sarcomere lengths (1.9 μm) active developed-forces and Ca^2+^-sensitivity, although stretching to 2.25 μm is associated with both force and Ca^2+^-sensitivity reductions in cardiac muscle preparations (Fukuda et al. 2001, 2005). Based on the latter, we speculate that at shorter sarcomere lengths, the normal capacity of tropomyosin to recruit functional units is less pronounced compared to longer sarcomere lengths due to less titin-induced strain on tropomyosin stiffness (i.e., persistence length). We recognize that trypsin digestion of titin at shorter muscle lengths have less effect on Ca^2+^-activated muscles. Titin-mediated tropomyosin-induced increases in stiffness would generate a long-range activation along the thin-filament (Fig. 2).

### In summary

the above data support the concept that titin contributes to the Frank–Starling “jigsaw”, in an all-at-once manner, following fiber lengthening. Titin appears to mediate length-dependent activation by synergistically affecting tropomyosin–troponin, and its long-range Ca^2+^-cooperative activation, but also by potentiating cross-bridge’s recruitment at the thick-filament with more Ca^2+^ bound on cTnC.

## Discussion

Over a century of research on the Frank–Starling Law, at the whole heart and the cardiomyocyte level, has vastly advanced our understanding of the fundamental basis of muscle. In particular, the elusive relationship between muscle lengthening and the sensitivity to Ca^2+^ ions appears to primarily involve the giant protein titin. It can regulate a composite of several synergistic processes that result in the formation of strongly-bound cross-bridges in thick-filaments that enhance Ca^2+^-affinity on cTnC. Not limited to, and open for debate, titin binding in the I-band region may increase the Ca^2+^-cooperative unit size by regulating the location (and persistence length) of tropomyosin and Ca^2+^-activation. Overall, titin mediation of length-dependent activation appears to: (1) orchestrate alterations in interfilament lattice spacing that approximate myosin and actin, and (2) induce strain in the thick-filaments that promotes the formation of strong-binding cross-bridges and cTnC alterations; while finally (3) titin-induced strain in the thin-filament increases the Ca^2+^-cooperative unit size.
